# Authors’ Response to Peer Reviews of “The Exchange of Informational Support in Online Health Communities at the Onset of the COVID-19 Pandemic: Content Analysis”

**DOI:** 10.2196/31329

**Published:** 2021-07-22

**Authors:** Wesley Jong, Ou Stella Liang, Christopher C Yang

**Affiliations:** 1 College of Medicine Drexel University Philadelphia, PA United States; 2 College of Computing and Informatics Drexel University Philadelphia, PA United States

**Keywords:** COVID-19, health information, informational support, online health, online health communities, online platform, pandemic, social support

This is the authors’ response to peer-review reports for “The Exchange of Informational Support in Online Health Communities at the Onset of the COVID-19 Pandemic: Content Analysis”.

## Round 1 Review

### Responses to Editors

M. Addressed [1].

Q. Addressed.

U. Addressed.

### Reviewer AB [2]

#### Specific Comments

##### Major Comments

1. Addressed.

2. Addressed.

3. Addressed.

4. Addressed.

5. Addressed. What we were trying to convey is that people who offered information were more likely to post more than once judging by their action of responding to others’ information requests.

6. Emotional support is an interesting topic, but it is out of the scope of this study.

7. We are unable to address this comment at this moment, as studies on other public health emergencies with comparable findings are limited.

##### Minor Comments

8. Addressed.

9. Addressed.

10. Addressed.

11. Addressed.

12. Addressed.

### Anonymous [3]:

#### General Comments

1. Addressed.

2. We are unable to address this comment at this moment.

3. Please refer to the *Methodology* section, where previous studies on which our coding ontology is based are cited.
